# Refractory Chronic Constipation in an Adolescent Female Later Diagnosed With Internal Anal Sphincter Achalasia

**DOI:** 10.7759/cureus.57135

**Published:** 2024-03-28

**Authors:** Farah Slaczka, Roshan Uruthirakumar, Mateusz Slaczka, Andrew Bozeman

**Affiliations:** 1 Pediatric Surgery, Trinity School of Medicine, Warner Robins, USA; 2 Pediatric Surgery, Fairview Park Hospital, Dublin, USA

**Keywords:** anal sphincter myectomy, botox injections, pediatric surgery, internal anal sphincter achalasia, chronic constipation, pediatric gastroenterology

## Abstract

Internal anal sphincter achalasia (IASA) is a rare anorectal disorder that presents as chronic refractory constipation in pediatrics. With a poor response to conventional constipation-based therapy, it is often misdiagnosed as other conditions, such as ultra-short-segment Hirschsprung disease. This case report describes a rare case of IASA in an adolescent female, emphasizing the importance of ruling out other differentials, including Hirschsprung disease, via rectal biopsy and thus allowing for earlier targeted therapy to improve lifestyle conditions. A 20-year-old female with a history of IASA presents for semiannual botulism toxin injections. Despite initial relief, her constipation symptoms gradually returned after four to five months. She has had a history of ineffective conventional constipation treatments since childhood, which prompted a further workup. Biopsy results during her teenage years confirmed the presence of ganglionic cells, differentiating IASA from Hirschsprung disease. The management plan involved biannual perianal Botox injections, offering relief for approximately six months. IASA's physiological basis involves altered innervation, the absence of nitrergic nerves, and defective neuromuscular junctions in the internal anal sphincter. Diagnosis requires anorectal manometry and a rectal suction biopsy. Treatment options include botulism, toxin injections, and posterior internal anal sphincter myectomy. Botulism injections offer temporary relief, while myectomy provides long-term improvement.

## Introduction

Anorectal disorders are relatively common in children, with constipation being the most prevalent. Initial presentations of constipation in children are typically handled conservatively with dietary changes, stool softeners, and laxatives. However, further exploration of the underlying condition is necessary when conventional treatment methods fail to resolve constipation. In a small subset of children with persistent constipation, the diagnosis of internal anal sphincter achalasia (IASA) has been made. This relatively rare condition is similar to Hirschsprung disease, with the major difference being the presence of ganglionic cells in the rectum. Due to the similarity in patient presentation, some cases of IASA have been misdiagnosed as ultra-short Hirschsprung and treated surgically [1].

IASA is a relatively understudied topic, and therefore, the prevalence and incidence of this condition are not widely known. One report cites the incidence as 4.5% among children investigated for chronic constipation [2]. A retrospective study done in 2020 reviewed 1,072 anorectal manometry studies (ARMs) done on children <18 years of age at the Nationwide Children’s Hospital between August 2010 and April 2019. Of these reviewed ARMs, 109 patients had an absent rectoanal inhibitory reflex (RAIR), which is the reflex that normally causes relaxation of the internal anal sphincter in response to distention of the rectum by gas or feces. Of these 109 patients with absent RAIR, 28 of them were diagnosed with IASA due to the presence of ganglion cells on rectal biopsy, giving a less than 3% diagnosis rate of IASA in this study [3]. This case report focuses on an adolescent female patient who has suffered from chronic constipation since childhood and received the diagnosis of IASA in her teenage years, with limited relief of symptoms from semiannual botulism injections.

This article was previously presented as a poster at the 2024 Georgia Association of Physicians of Indian Heritage (GAPI) Conference on March 9, 2024.

## Case presentation

A 20-year-old female with a history of IASA presented to the pre-operative area of the hospital in June 2023 for her semiannual botulism toxin injection procedure. She last underwent Botox treatment in December 2022, after which she had significant relief from her constipation. However, over the last month, she has noticed an increasing return of her constipation symptoms, with her last bowel movement being six days ago. Laxative therapy with Dulcolax and MiraLax has provided her with no relief in the past few weeks. She has no other medical history and has complained of no other symptoms.

The patient was initially referred for her symptoms of severe, refractory constipation to the Children’s National Colorectal Center for further workup and diagnosis in December 2020. At that time, she was on Bisacodyl 20 mg and MiraLax 34 mg daily, which enabled her to have a bowel movement approximately twice a week. In January 2021, she underwent colonic and anorectal manometry with anesthesia and Botox injection therapy. Post-Botox treatment, she was able to have a bowel movement every other day. She was also advised to take two tablets of 10 mg of Dulcolax, which may be increased to three tablets the next day if constipation persists.

**Figure 1:**
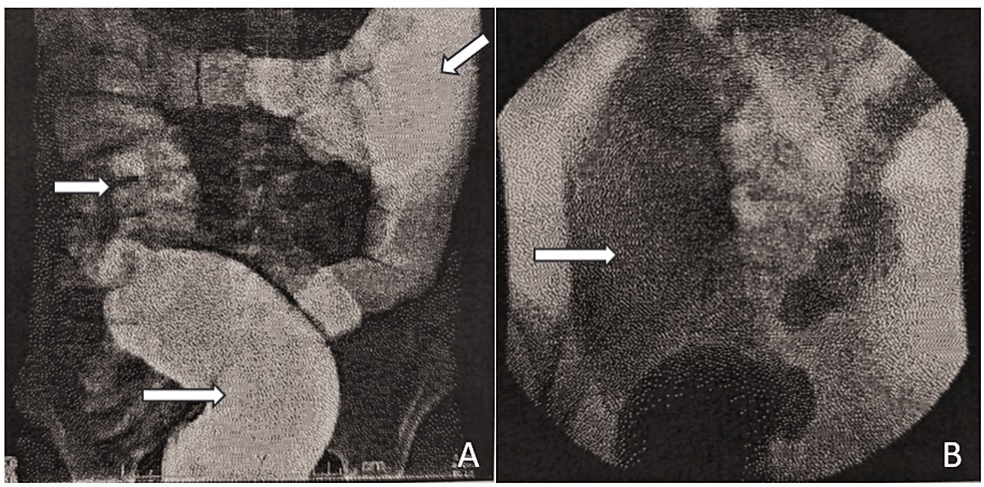
Contrast enema of colon and sigmoid colon Contrast enema showed an enlarged colon and sigmoid colon (A). No transition zone was identified to suggest Hirschsprung disease. No clear obstructive lesions or strictures were seen. Post-evacuation film (B) showed good evacuation of contrast from the colon. These findings are suggestive of IASA by eliminating other potential causes of chronic constipation.

In March 2021, she underwent contrast enema of the colon, which revealed a large colon and sigmoid colon with no transition zone identified, which ruled out a diagnosis of Hirschsprung disease (Figure 1). In a follow-up visit with the Colorectal Center in April 2021, she described a gradual return of her constipation symptoms, with her last bowel movement being 10 days ago. The colorectal surgeon and gastrointestinal motility specialist reviewed her medical history and manometry results and concluded that she has overall normal colonic manometry, but a rectoanal inhibitory reflex was inconsistently seen. They recommended that a biopsy be performed to make a diagnosis of internal anal sphincter achalasia.

A full-thickness rectal biopsy was performed in June 2021 by her local pediatric surgeon. The biopsy was approximately 1-1.5 cm proximal to the dentate line, extending proximal towards the rectum. The specimen was secured to a tongue depressor and labeled proximal and distal. The specimen was sent to pathology for gross and microscopic analysis. Results of the biopsy showed the presence of ganglionic cells, ruling out Hirschsprung disease and leading to a diagnosis of internal anal sphincter achalasia.

She has continued therapy with four quadrant injections of 100 units total of Botox (25 units in each quadrant) at her local hospital to treat her symptoms of intractable constipation. Each round of injections has provided her with relief of symptoms for approximately six months, with the gradual return of constipation. Between injections, she takes two tablets of Dulcolax orally every evening but will still go several days to a week without a bowel movement. She has had episodes of severe abdominal distention with associated pain as a result.

## Discussion

Davidson and Bauer first described internal anal sphincter achalasia in 1958, reporting three cases of constipation from birth without a zone of narrowing on barium enema examination. They also examined histological specimens of the colon and identified the presence of ganglia in all regions of the colon. They, therefore, described this phenomenon as “achalasia of the rectal distal segment.” Over time, this terminology eventually changed to “ultra-short segment Hirschsprung disease” and finally to “internal anal sphincter achalasia” [4].

It is essential to understand the physiology and innervation of the internal anal sphincter to fully appreciate what occurs in IASA. The internal anal sphincter is crucial for fecal continence and responds to rectal distention via the rectosphincteric inhibitory reflex. Nitric oxide is a significant mediator that causes relaxation of the internal anal sphincter in response to adequate rectal distention. IASA may be the result of several pathological issues, including altered innervation, absence of nitrergic nerves, defective neuromuscular junctions, and abnormal distribution of interstitial cells of Cajal (ICC), which are important for electrical pacemaker activity and neurotransmission. The absence of ICC may result in impaired nitric-oxide-mediated signaling, resulting in a lack of relaxation to the internal anal sphincter. In one study done by Piotrowska et al. in 2003, they found that normal internal anal sphincter samples contained abundant and uniformly distributed peripherin-immunoreactive nerve fibers as well as a large number of ICC cells. In contrast, samples of the internal anal sphincter in patients with IASA have markedly reduced peripherin-immunoreactive nerve fibers and ICCs, while these nerve fibers and ICCs were absent or sparse in Hirschsprung disease patient samples [5]. Therefore, to make a definitive diagnosis of IASA, anorectal manometry and a rectal suction biopsy are required to rule out other potential causes of chronic constipation in children [1].

Treatment options are currently limited to four-quadrant botulism toxin injections and posterior internal anal sphincter myectomy. As was described previously in this case report, the patient only found relief for her constipation and abdominal bloating symptoms after receiving botulism toxin injections. While she schedules these injections biannually, she only finds significant relief of symptoms for approximately four months after the injections, after which she has a gradual return of constipation and bloating despite the use of various laxatives. There are several risk factors associated with botulism toxin injections, including urinary incontinence, pelvic muscle paresis, perianal abscess formation, pruritis ani, and rectal prolapse [6]. While the occurrence of these adverse effects is relatively rare, they are still important aspects to consider when deciding upon an appropriate treatment plan for patients with IASA.

The definitive treatment for IASA is posterior internal anal sphincter (IAS) myectomy. In a study done in 2000 by Caluwe et al., 15 patients between the ages of 2 and 12 with IASA underwent myectomy of the internal anal sphincter. Of these patients, seven had regular bowel movements without the use of laxatives, six stayed regular on low-dose laxatives, one required regular enemas to stay regular, and one required resection of the dilated redundant sigmoid colon but has had regular bowel habits since then [6]. In 2012, Friedmacher and Puri conducted a meta-analysis of 16 prospective and retrospective studies published between 1973 and 2009 to compare the effectiveness of botulism toxin injections versus myectomy. They concluded that the rate of transient fecal incontinence was significantly higher after Botox injections, as were subsequent surgical treatments. Short- and long-term improvements were significantly more frequent after IAS myectomy [7]. However, as some of the detrimental effects of IAS myectomy, such as fecal incontinence, rectal bleeding, rectal urgency, and wound healing issues, may not become apparent for several years after the procedure, many pediatric surgeons avoid performing this procedure for as long as possible [3], as is the case with this patient. Due to her young age and active lifestyle, she did not want to risk having side effects such as fecal incontinence and has so far deferred treatment with a myectomy in preference to continuing botox injection treatment.

In the retrospective study done in 2020, the researchers noticed that IASA typically had a later onset of symptoms, likely had normal meconium passing at birth, and was diagnosed later in childhood compared to Hirschsprung's disease patients [3]. It is, therefore, essential to perform a thorough workup on children presenting with a history of chronic constipation refractory to traditional treatments of lifestyle and diet modifications. This would help to avoid cases of rarer conditions, such as IASA, going undiagnosed or being misdiagnosed and treated incorrectly. Once a diagnosis of IASA is made, the pros and cons of all treatment options should be discussed with the patient and family to decide the best course of action for that particular patient. With more education about this topic, we can hope to identify more cases of IASA and learn about the effectiveness of the various treatment methods in providing these patients with a more comfortable lifestyle.

## Conclusions

In elucidating the intricacies of IASA, this case underscores the critical diagnostic challenges in distinguishing IASA from its phenotypically similar counterpart, Hirschsprung's disease. The comprehensive exploration of the patient's history, diagnostic trajectory, and treatment response reveals the nuances involved in managing this rare anorectal disorder. Our patient, a 20-year-old female, manifested a recurrent pattern of constipation refractory to conventional therapies, triggering an in-depth investigation culminating in the diagnosis of IASA. The pivotal role of rectal biopsy in confirming the presence of ganglionic cells serves as a diagnostic necessity, effectively differentiating IASA from Hirschsprung's disease. This diagnostic precision holds paramount importance, given the therapeutic differences between these conditions.

Therapeutically, the case displays the temporality of relief associated with botulism toxin injections, providing a deeper understanding of the treatment's efficacy and its limitations. The subsequent consideration of posterior internal anal sphincter myectomy as the definitive treatment underlines the gravity of decision-making in managing IASA, with careful weighing of risks and benefits. Furthermore, the insights garnered from this case contribute to the evolving landscape of pediatric anorectal disorders. The learning objectives encompass not only the recognition and diagnosis of IASA but also the judicious selection of treatment modalities guided by individual patient characteristics. As we navigate the subtleties of IASA management, this case underscores the need for ongoing research and education so more pediatric patients will benefit from early diagnosis and management of such disorders to improve patient quality of life as soon as possible.
